# A comprehensive study of Al-Cu-Mg system reinforced with nano-ZrO_2_ particles synthesized by powder metallurgy technique

**DOI:** 10.1038/s41598-024-53061-9

**Published:** 2024-02-04

**Authors:** Essam B. Moustafa, Abdulrahman Aljabri, Waheed S. Abushanab, E. Ghandourah, Mohammed A. Taha, Ahmed B. Khoshaim, Rasha A. Youness, S. S. Mohamed

**Affiliations:** 1 Mechanical Engineering Departments, Faculty of Engineering, King Abdulaziz University, 21589 Jeddah, Saudi Arabia; 2 Department of Mechanical Engineering, Islamic University of Madinah, 42351 Medina, Saudi Arabia; 3 Marine Engineering Department, Faculty of Maritime Studies and Marine Engineering, King Abdulaziz University, 21589 Jeddah, Saudi Arabia; 4 Department of Nuclear Engineering, Faculty of Engineering, King Abdulaziz University, 21589 Jeddah, Saudi Arabia; 5 Solid State Physics Department, National Research Centre, El Buhouth St., Dokki, Giza, 12622 Egypt; 6 Spectroscopy Department, National Research Centre, El Buhouth St., Dokki, Giza, 12622 Egypt; 7 Mechanical Engineering Department, Shoubra Faculty of Engineering, Benha University, Cairo, Egypt

**Keywords:** Materials science, Nanoscale materials

## Abstract

More focus has recently been placed on enhancing the strength, elastic modulus, coefficient of thermal expansion (CTE), wear and corrosion resistance, and other qualities of aluminum (Al) alloys by varying the quantity of ceramics added for a range of industrial uses. In this regard, Al-4.2-Cu-1.6Mg matrix nanocomposites reinforced with nano-ZrO_2_ particles have been created using the powder metallurgy approach. The microstructure and particle size distributions of the produced powders were analyzed using a diffraction particle size analyzer, XRD, TEM, and SEM. To achieve good sinterability, the powders were compacted and sintered in argon. The sintered nanocomposites' mechanical, elastic, and physicochemical characteristics were measured. Additionally, the behavior of corrosion, wear, and thermal expansion were examined. The results showed a decrease in the particle sizes of the Al-Cu-Mg alloy by adding ZrO_2_ nanoparticles up to 45.8 nm for the composite containing 16 wt.% ZrO_2_. By increasing the sintering temperature to 570 °C, the densification of nanocomposites was enhanced. Also, the coefficient of thermal expansion and wear rate remarkably decreased by about 28 and 37.5% by adding 16 wt.% ZrO_2_. Moreover, microhardness yield, strength, and Young’s modulus were enhanced to 161, 145, and 64%, respectively, after adding 16 wt.% ZrO_2_. In addition, increasing the exposure time was responsible for decreasing the corrosion rate for the same sample.

## Introduction

In recent years, technological progress has required unique properties such as high strength, high corrosion resistance, better fatigue strength, high wear resistance, etc., which cannot be achieved in metals alone. In this context, researchers have considered manufacturing metal-based composites. Aluminum (Al) is the best-preferred material as a matrix for Al matrix compounds' production (AMCs) reinforced with ceramic particles. Due to these attractive properties, AMCs have a strong role in different industrial areas such as car enterprises, aerospace, defense, and military industries^1–4^. On the other hand, Al alloys are best used as a matrix due to their high strength and low ductility compared to pure Al.

Notably, several articles attribute Al and its alloys' high resistance against corrosion in air to their ability to form an oxide layer that protects them from the attack of corrosive solutions. To further improve their corrosion resistance, some corrosion inhibitors can be added to modify the neighboring environment. Notably, these corrosion inhibitors include alloying elements, anodizing the surface, and painting their surfaces with a protective coating layer^5–7^. In spite of these amazing properties, other monolithic alloys are more desirable than AMCs, where the latter nanocomposites possess several drawbacks such as manufacturing defects, internal stress, differences in microstructure, and coupling of the matrix and reinforcement, which consequently leads to galvanic effects^8^. Several studies are investigating the properties of Al and Al alloy matrix composites. For example, Zulfia et al.^4^ used the Compo casting method to produce Al7010 alloy reinforced with ZrO_2_ particles. The results showed a clear improvement in the composite's tensile strength and hardness of about 26 and 46%, respectively, by adding 8 vol.% of SiC particles. ZrO_2_ nanoparticles were mechanically alloyed with Al2024 alloy powder by Youness et al.^9^ to create nanocomposite powders with varying ZrO_2_ concentrations. Following that, they underwent argon atmosphere pressing and sintering to get bulk nanocomposite samples. By adding more ZrO_2_, the nanocomposites’ microhardness and compressive strength were greatly enhanced. Muralidharah et al.^10^ studied the effect of different ZrO_2_ weight percentages up to 12 wt.% on the mechanical properties of the Al 6061 alloy using stir casting. It was nanocomposites that observed an improvement in mechanical properties by adding ZrO_2_ particles. AbuShanab et al.^11^ extensively studied the effect of graphene weight percent on Al2024 alloy matrix nancomposites using powder metallurgy methods. Their results showed graphene's effect on microstructure, mechanical and thermal expansion, corrosion behavior, and these metal matrixes’ electrical properties. Importantly, the addition of ceramics like SiC^12^, Al_2_O_3_^13^, ZrO_2_^4,9,10,14^, TiC^15^, and graphene^6^ is considered the optimal solution to overcome this serious obstacle. In this sense, finely stabilized ZrO_2_ is a good choice for this required task due to its superior mechanical properties, high melting temperature, better corrosion resistance, and perfect corrosion resistance compared to Al and its alloys' thermal and chemical stability^4,16^. Under the effect of its particle size, the interaction of particles with dislocations easily occurs, which consequently has a great significance for the enhancement of densification of the sintered samples and, consequently, the improvement of wear, corrosion resistance, and mechanical properties^17^.

It is important to emphasize that there are many effective methods to produce Al and its alloy matrix nanocomposites, such as fraction stir^18^, squeeze casting^19^, stir casting^20^, and powder metallurgy (PM)^21,22^. The latter is a modern and cost-effective tool for good dispersion of reinforcement in the matrix, giving a uniform distribution of particles in the microstructure^23,24^. Due to these benefits, the mechanical alloying (MA) technique has been used as one of the most effective PM methods to prepare desired nanocomposites. During the MA process, powders undergo a fracturing and welding process for powder particles that is highly dependent on key parameters such as milling time, speed, mill type, ball-to-powder (BPR) ratio, and vial/ball material, which thus control the size and shape of the reinforcement material produced^25^.

Based on the abovementioned advantages of PM, this process can fabricate various MMCs with desirable properties^26^. In previous research, the effect of different ceramics, including ZrO_2_, on some properties of Al-Cu-Mg alloys prepared by different methods such as stir castand others, was studied, and this is the reason why the use of aluminum alloys is restricted in many industries. Based on these facts, the goal is an extensive study to improve the mechanical and elastic properties, the coefficient of thermal expansion, and its resistance to abrasion and corrosion by adding different percentages of nano-ZrO_2_ particles, up to 16% by weight, prepared by powder metallurgy.

## Materials and experimental setup

In this work, we selected an Al–4.2 Cu–1.6Mg Al alloy as a matrix, and nano-ZrO_2_ (< 50 nm) particles used as reinforced with various weight percentages up to 16 wt.%. The mechanical alloying method used to prepare the Al–4.2Cu–1.6Mg alloy, the following commercial materials have been used: Al (99.9 wt.%), Cu (99.9 wt.%), and Mg (99.95 wt.%). Al alloy components have been blended with a planetary ball mill for 20 h with rotation speed = 120 rpm. In order to obtain nanocomposites, the nano-ZrO_2_ was added with different weight percentages to the Al–10Si–0.6Mg alloy shown in Table 1. Subsequently, these mixtures were subjected to the a milling process for 20 h with rotation speed = 500 rpm having in mind that the milling process was done in a cycle of 2 h and paused for 2 h.

**Table 1:** The composition of the prepared samples.

Sample	The composition (wt. %)	
	Al-Cu- Mg alloy	ZrO_2_
AZ0	100	0
AZ2	98	2
AZ4	96	4
AZ8	92	8
A16	84	16

To investigate the morphology of the mechanically alloyed powders, they were characterized using X-ray diffraction (XRD), transmission electron microscopy (TEM, type JEOL JEM-1230) and particle size was measured using a diffraction particle size analyzer to get the average distribution pattern for each powder. According to in our work^6^, the crystallite size of milled powders was calculated depending on the broadening of the diffraction peaks using the Scherrer equation. Then, the milled powders were pressed and sintered at 470 and 570 °C in an argon atmosphere for 1 h. Notably, the rule of mixture was carried out to calculate the theoretical densities of samples taking into account the density of the Al-Cu-Mg alloy = 2.7 g/cm^3^ and the density of ZrO_2_ = 5.68 g/cm^3^. On the other hand, Archimedes method was carried out to measure both bulk density and apparent porosity. The sintered samples' microstructure was investigated by scanning electron microscopy (SEM; Philips XL30). Moreover, using (Netzsch DIL402 PC; Germany), thermal expansion of specimens was measured in the range from 30 to 400 °C. Vickers microhardness (HV) was measured with a Shimadzu-HMV (Japan) according to ASTM: B933-09 as described in Ref.^27^. Furthermore, the compressive tests of the sintered nanocomposites were performed according to ASTM E9–19 standard. The ultimate strength, yield strength, and elongation were calculated from the stress–strain curve; hence the ultimate strength and elongation are the maximum values of stress and strain on stress–strain curve, respectively. On the other hand, yield strength was calculated using the 0.2% offset principle. Using the pulse-echo technique system, the longitudinal and shear velocities of the ultrasonic wave were obtained at room temperature to obtain Elastic moduli as indicated from Ref.^28–30^. The wear test was carried out using a pin-on-disk tester machine; the specimens were weighed and measured by a digital balance of accuracy of 0.0001 g. All samples were prepared with the same dimensions and polished well using grinding papers with different grades (600 to 4000). The test was carried out using four different loads. The wear rate due to the weight loss was calculated from the following equations (Eqs. 8 and 9)^31^:

1 $$ {\text{Net weight }} = {\text{ weight before wear }} - {\text{ weight after wear}}, $$

2 $$ {\text{Wear rate }} = {\text{ net weight}}/{\text{time}}{.} $$

The sintered samples’ corrosion rate was determined using static immersion weight loss method at room temperature where each sample was weighed before its immersion in 1 M HCL solution and later taken out after 24, 48, 72, 96, 120, 144, and 168 h. After drying thoroughly, the specimens were weighted again. The weight loss was measured and converted into corrosion rate expressed in mm penetration per year (mm/year). The standard deviation of all measured propertes was calculated for 5 samples.

## Results and discussion

### Milled powders

**Figure 1:**
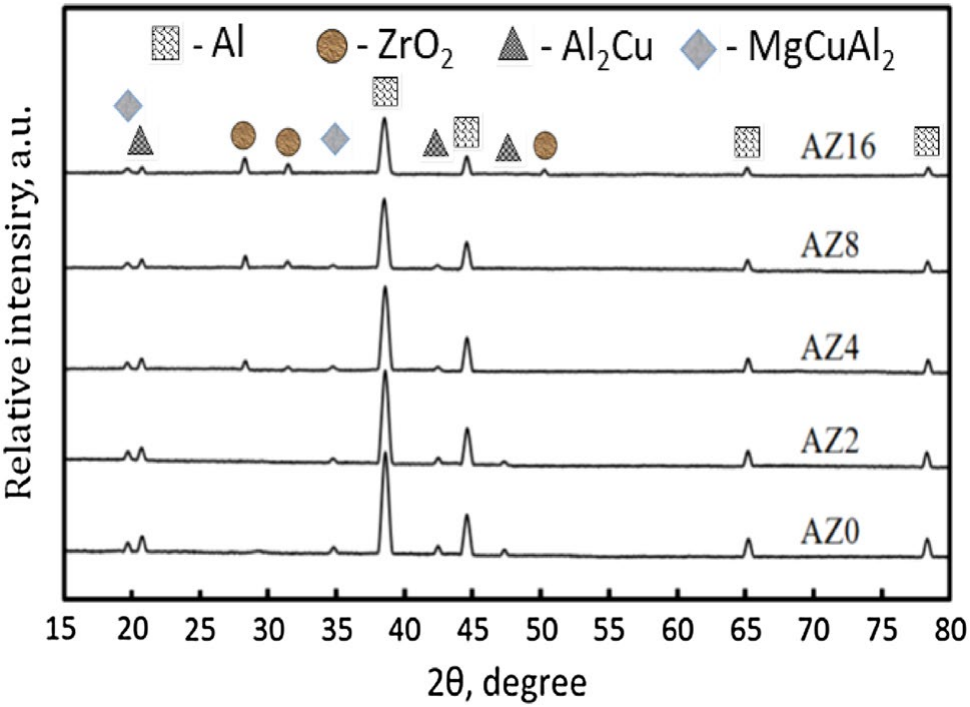
The XRD patterns of the AZ0, AZ2, AZ4, AZ8 and AZ16 milled powders.

**Figure 2:**
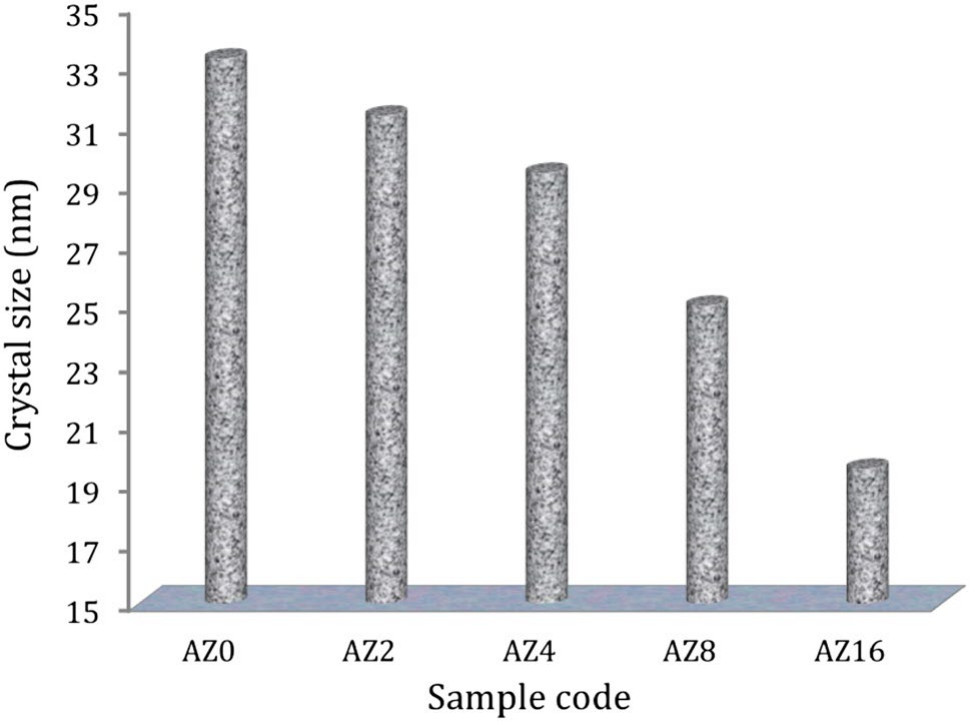
Effect of adding ZrO_2_ particle on the crystal size of milled powders.

Figure 1 shows the XRD patterns of AZ0, AZ2, AZ4, AZ8 and AZ16 nanocomposites powder after 20 h of milling. The XRD pattern of sample AZ0 (matrix) clearly shows that, in accordance with the card numbers (JCPDS 01-089-4037, 96-901-2197 and 65-2501), the powder’s primary phases are Al, Al_2_Cu and MgCuAl_2_, respectively. Cu’s solid solubility in the solid Al solution phase caused the Al_2_Cu (precipitation) phase to form, confirming that the alloying process had been effectively completed in accordance with the phase diagram. On the other hand, ZrO_2_ peaks appear as a result of its addition in different percentages according to card numbers (JCPDS 86-1450). It is possible to conclude that when ZrO_2_ weight percent gradually rises, their broadness increases and their intensities noticeably decrease, which leads to a decrease in the size of the crystals, as shown in Fig. 2. The mean crystal sizes value of the AZ0, AZ2, AZ4, AZ8, and AZ16 specimes were 33.25, 31.34, 29.42, 24.98, and 19.52 nm, respectively.

**Figure 3:**
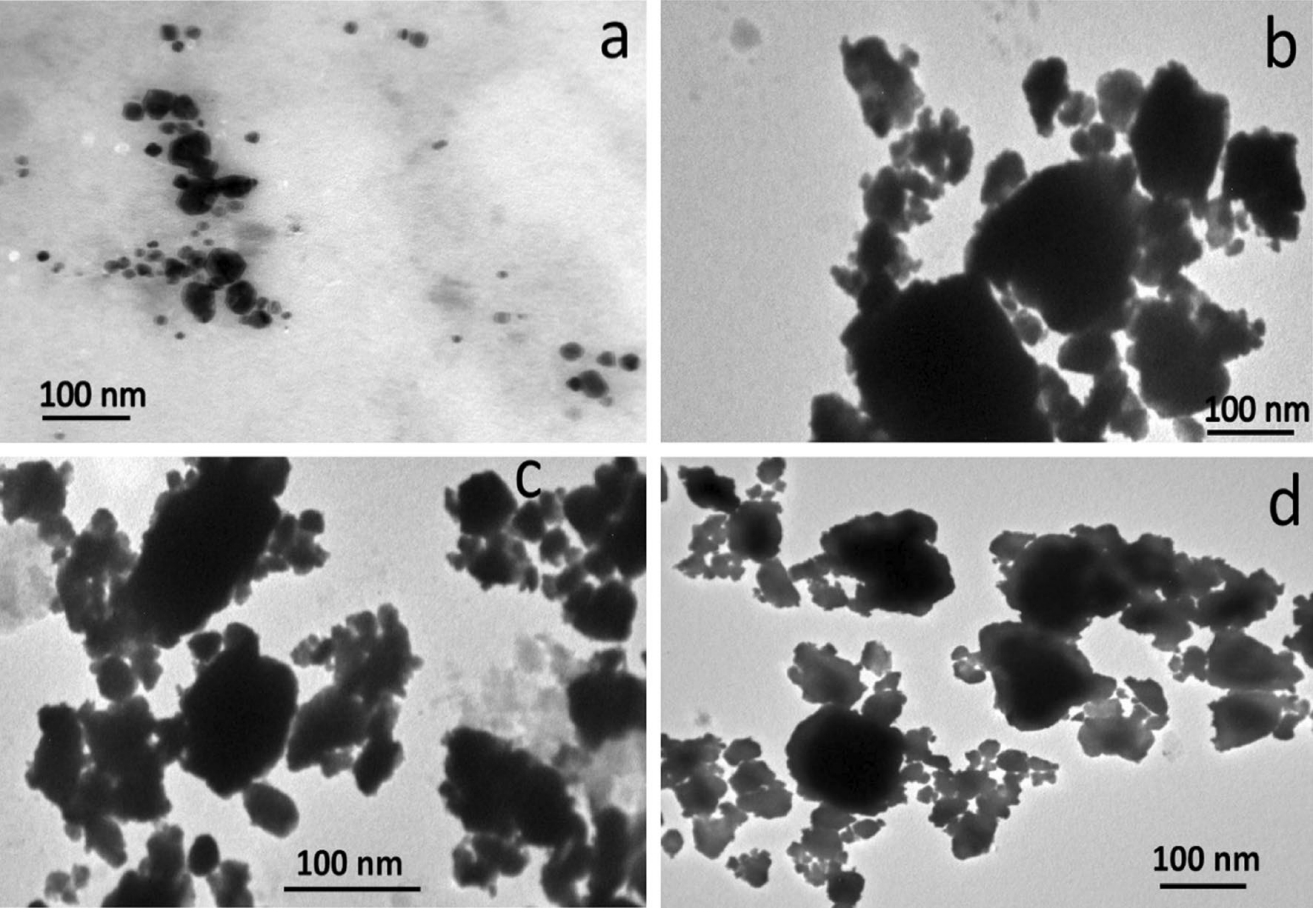
TEM micrographs of (**a**) as-received ZrO_2_ nanoparticles, (**b**) AZ0, (**c**) AZ8, and (**d**) AZ16 samples.

**Figure 4:**
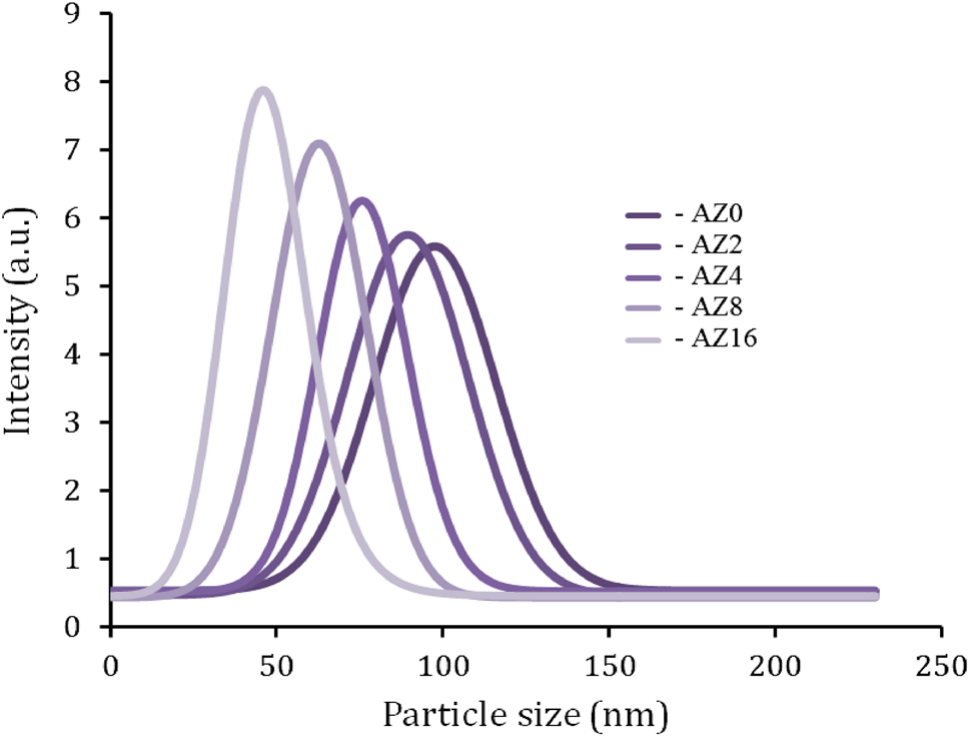
Particle size distribution of the milled powders in various weight percent of ZrO_2_ particle.

TEM micrographs of as-received ZrO_2_ nanoparticles along with those of the Al alloy containing 0, 8, and 16 wt.% ZrO_2_ after milling are illustrated in Fig. 3a–d. As can be seen from Fig. 3a, ZrO_2_ particles are a little agglomerated, located in the nano-scale range, and their sizes do not exceed 50 nm. The most plausible explanation for these data is that, although the ZrO_2_ particles are fragmented during mechanical milling, the Al alloy matrix particles undergo deformation (flatting). ZrO_2_ particles sandwich one or more matrix particles at the point of ball contact as the ductile Al alloy matrix particles begin to weld. Real nanocomposite powders are created as a result of ZrO_2_ particles residing at the interfacial boundaries of the welded matrix particles^6^. It is noteworthy to observe that the local plastic deformation around the ZrO_2_ particles has increased, contributing to the particle size reductions. Additionally, ZrO_2_ particles can result in increased energy transmission to the Al alloy matrix by acting as milling balls. On the other hand, with increasing ZrO_2_ contents, a fracture-cold welding mechanism quickly occurs^8^. Figure 4 shows the particle size distribution of the all milled powders. As the ZrO_2_ weight percent increased, the particle size decreased and the distribution shifted to smaller sizes. The mean particle sizes value of the AZ0, AZ2, AZ4, AZ8, and AZ16 specimes were 97.7, 89.3, 79.4, 62.1, and 45.8 nm, respectively.

### Sintered nanocomposites

#### Physical properties

**Figure 5:**
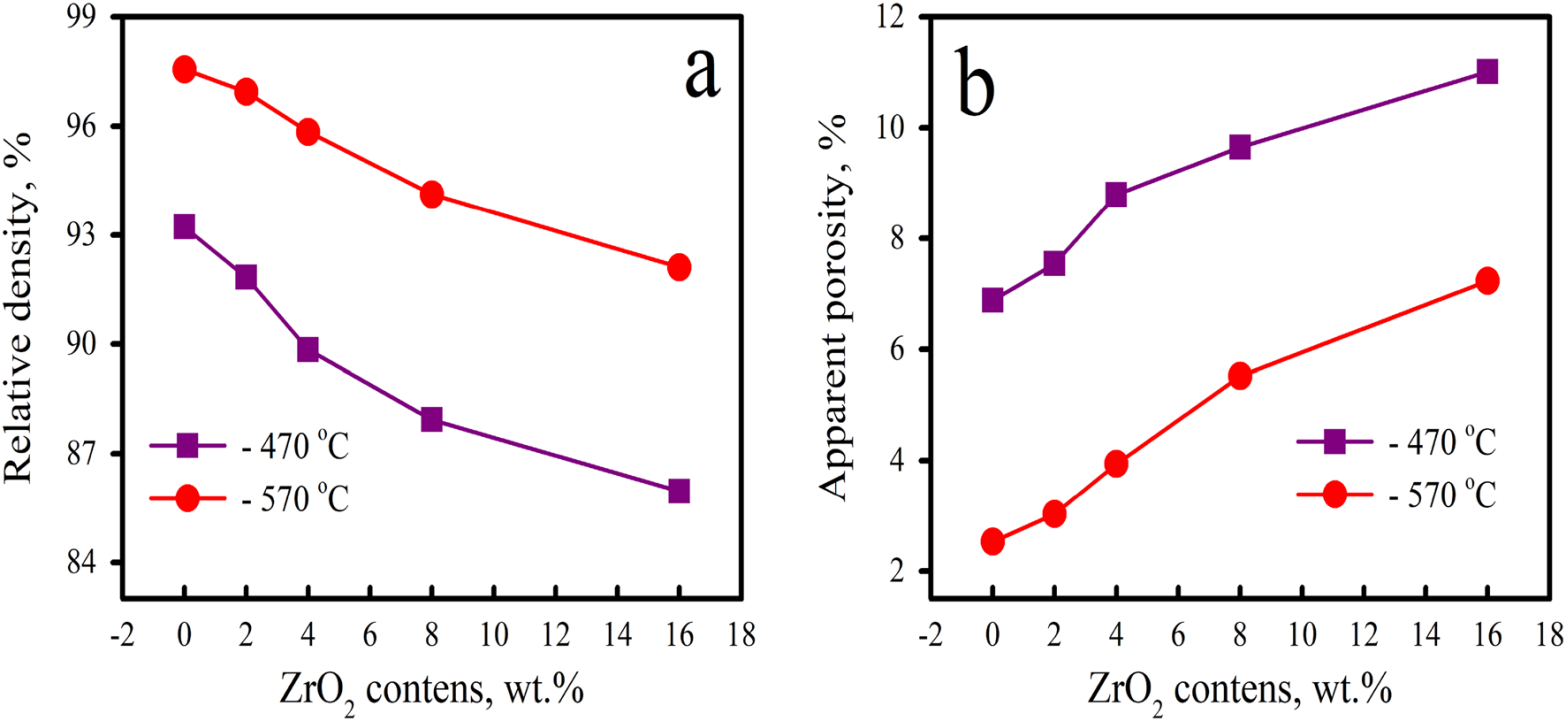
(**a**) Relative density and (**b**) apparent porosity of the sintered samples.

After the mechanical alloying process, the compaction of the milled nanocomposite powders is often an important step in obtaining bulk materials. Consequently, this stage regulates the final sintered nanocomposites’ porosity and form^32^. Figure 5 shows the bar graph that corresponds to the ZrO_2_ weight percentages and describes the relative density and apparent porosity of the sintered samples during 1 h at 470 and 570 °C. Furthermore, the standard deviation of the measured relative density and apparent porosity are listed in Table 2. The theoretical densities of AZ0, AZ2, AZ4, AZ8, and AZ16 samples = 2.65, 2.68, 2.71, 2.77, and 2.90 g/cm^3^, respectively. The relative density values of AZ0 and AZ16 after sintering at 460 °C are 93.22 and 85.96%, respectively. Conversely, the apparent porosity values are 6.88 and 11.01%, respectively, for the same samples sintered at 460 °C. This can be the result of the higher hardness of the ZrO_2_ ceramic particles in the Al alloy matrix, which reduces the pressing capacity of the sintered samples as ZrO_2_ weight percentages rise. Additionally, ZrO_2_ reinforcement has a melting temperature that is around 2715 °C, which is substantially higher than that of the Al matrix. As a result, higher ZrO_2_ levels impede the sintering process and function as a barrier against diffusion steps during this process^33^. Conversely, raising the sintering temperature from 470 to 570 °C can effectively improve relative density because it causes more necks to develop between particles and increases the amount of bonding between them^34^. Furthermore, faster solid-state diffusion and improved densification behavior are the results of higher sintering temperatures^35^. By increasing the ZrO_2_ contents from 0 to 16 weight percent, the relative density of samples drops from 97.56 to 92.11% when the sintering temperature is equal to 570 °C.

**Table 2:** The standard deviation of relative density and apparent porosity for all samples tested.

Sample	Standard deviation			
	Relative density		Apparent porosity	
	470 °C	570 °C	470 °C	570 °C
AZ0	0.232	0.243	1.70624	0.60
AZ2	0.222	0.221	1.822418	0.69
AZ4	0.211	0.217	2.063892	0.87
AZ8	0.174	0.181	1.909735	1.02
AZ16	0.132	0.145	1.691136	1.04

#### Microstructure

**Figure 6:**
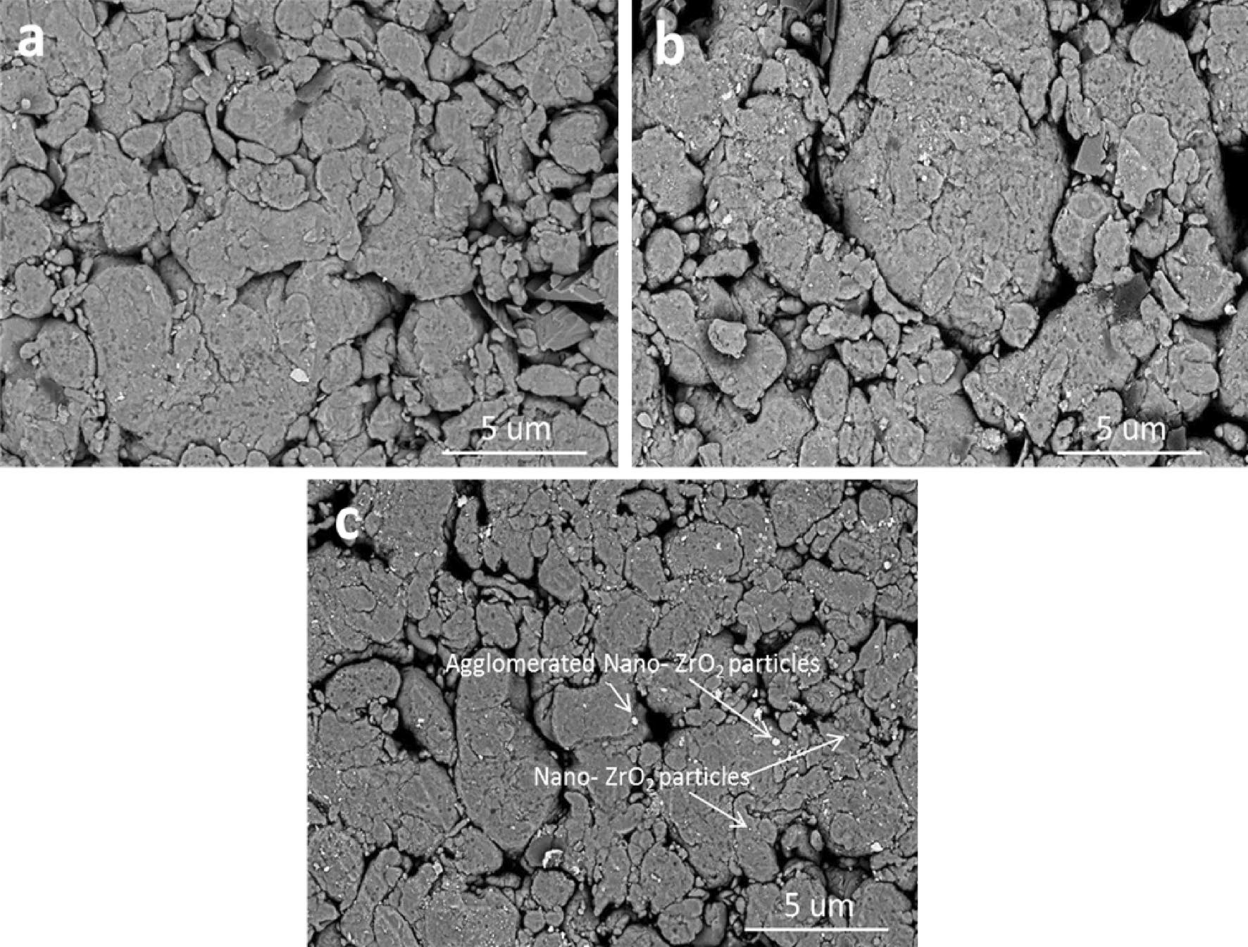
SEM images of (**a**) AZ0, (**b**) AZ4 and (**c**) AZ16 compacted samples.

**Figure 7:**
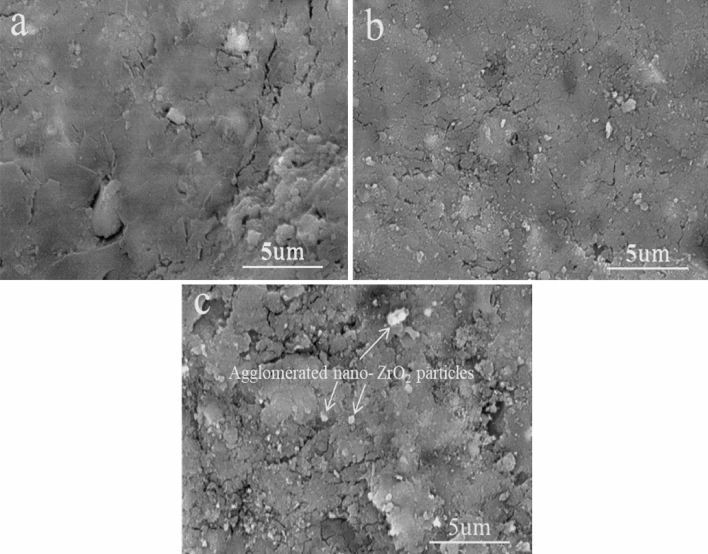
SEM micrographs of (**a**) AZ0, (**b**) AZ4 and (**c**) AZ16 samples sintered at 470 °C.

**Figure 8:**
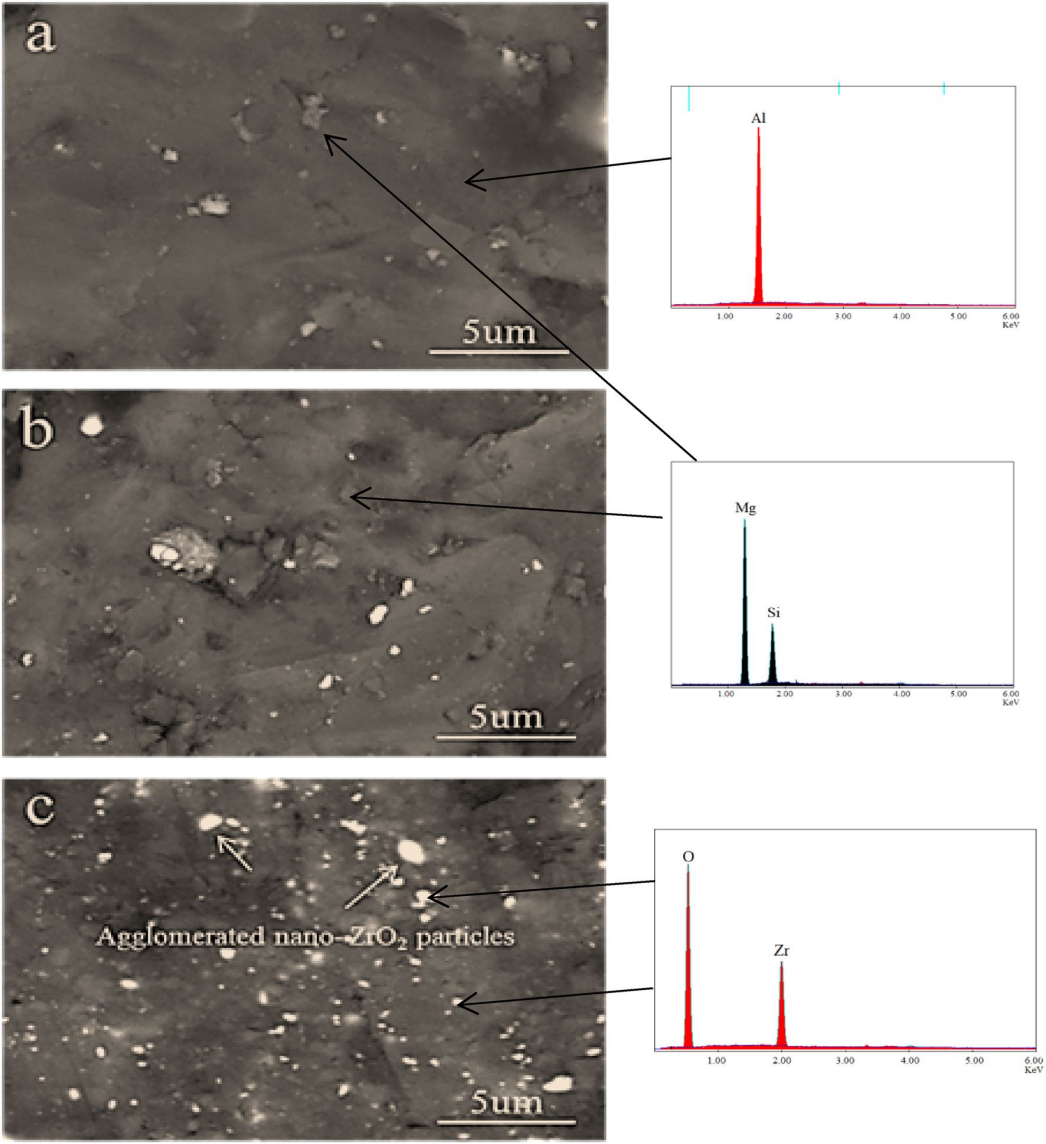
SEM micrographs of (**a**) AZ0, (**b**) AZ4, and (**c**) AZ16 samples sintered at 570 °C along with their corresponding EDS patterns.

SEM micrographs of the compacted AZ0, AZ4, and AZ16 nanocomposites powders, after 20 h of milling and compressing at 50 MPa, are shown in Fig. 6a–c. Careful analyses of SEM micrographs reveal that the distribution of nano-ZrO_2_ particles in Al alloy matrix with good densification has a strong role in the mechanical and electrical properties of the final nanocomposites. Figures 7 and 8 illustrated the SEM images of nanocomposites with different nano-ZrO_2_ contents and sintered at 470 and 570 °C along with their corresponding EDS patterns. Generally, at the lower sintering temperature, i.e. 470 °C, nano-ZrO_2_ particles are found at the Al alloy matrix's grain borders, considering the sample has the lowest ZrO_2_ content exhibits homogenous distribution for ZrO_2_ particles noting that this good distribution decreases with increased ZrO_2_ contents. Accordingly, the distribution of ZrO_2_ particles in both AZ4 nanocomposites specimens is homogenous, while that of AZ16 samples decreases. Notably, porosity shows the reverse pattern, increasing as ZrO_2_ particle concentration in the specimens under study increases. Nevertheless, greater densification behavior that is, almost attaining full density is produced by raising the sintering temperature to 570 °C, which promotes the diffusion process throughout the heating phase. During the sintering of the nanocomposites samples, the contact border between the particles appears to be expanding, signifying the attainment of strong nano-ZrO_2_-matrix interfacial bonding and the absence of pores in the ZrO_2_ particle area.

#### Thermal expansion behavior

**Figure 9:**
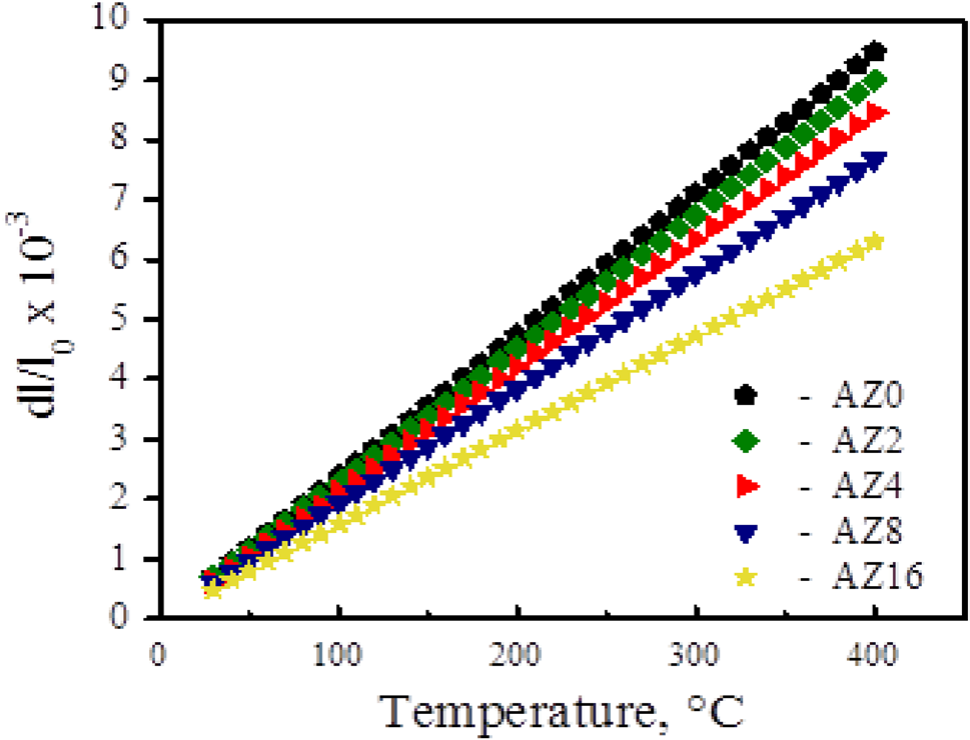
Thermal expansion behavior of samples versus ZrO_2_ contents.

**Figure 10:**
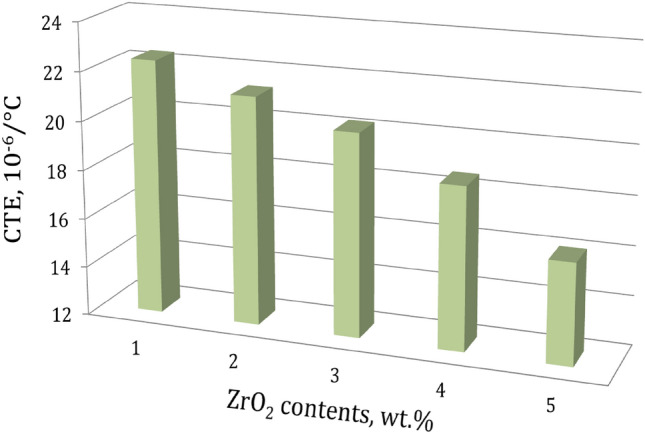
The changes in CTE values versus ZrO_2_ contents of the sintered nanocomposites.

Figure 9 depicts the relative thermal expansion (Δl/l) behavior of the sintered Al alloys and nanocomposite samples in the temperature range of 30–400 °C. In the specified temperature range, the un-reinforced Al alloy matrix has a higher Δl/l range from 0.679 × 10^–3^ to 9.97 × 10^–3^ compared with 0.495 × 10^–3^ to 6.45 × 10^–3^ for the composite containing 16 wt.% of ZrO_2_ particles (AZ16) The variations in CTE of the sintered samples, as determined by the slope of the thermal expansion curve (Fig. 9), are shown in Fig. 10. It is evident that lower CTE values are caused by higher ZrO_2_ concentrations. On the other hand, CTE values rise as the sintering temperature rises. As anticipated, the CTE of the nanocomposite samples is greatly decreased when ZrO_2_ particles are added to the Al alloy matrix. This conclusion is supported by the fact that ZrO_2_’s CTE is lower than the Al alloy matrix's, thereby limiting Al’s thermal expansion and improving the Al alloy matrix's dimensional stability^36^. However, because the CTE values of the ZrO_2_ particles and the Al alloy matrix do not match, the addition of ceramic reinforcements (ZrO_2_ particles) to the metal matrix (Al alloy) results in residual stresses in the matrix. The Al alloy matrix experiences plastic deformation as a result of thermal stresses, and this process is crucial for enhancing the strength of nanocomposites^37^.

#### Elastic and mechanical properties

**Figure 11:**
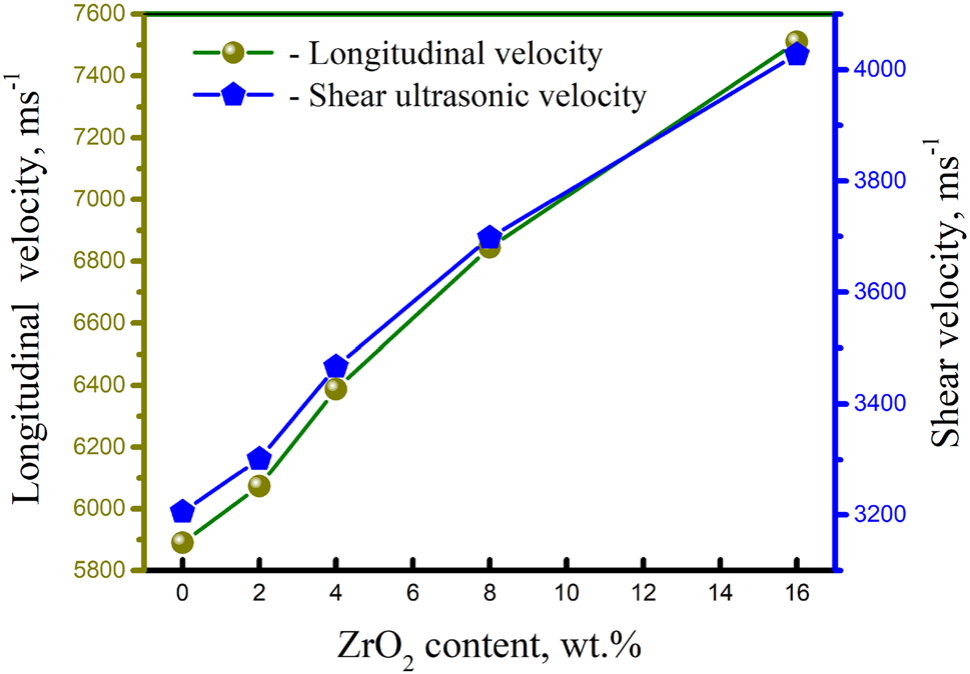
Ultrasonic velocities of nanocomposites samples sintered at 570 °C in relation to varying ZrO_2_ weight percent.

**Figure 12:**
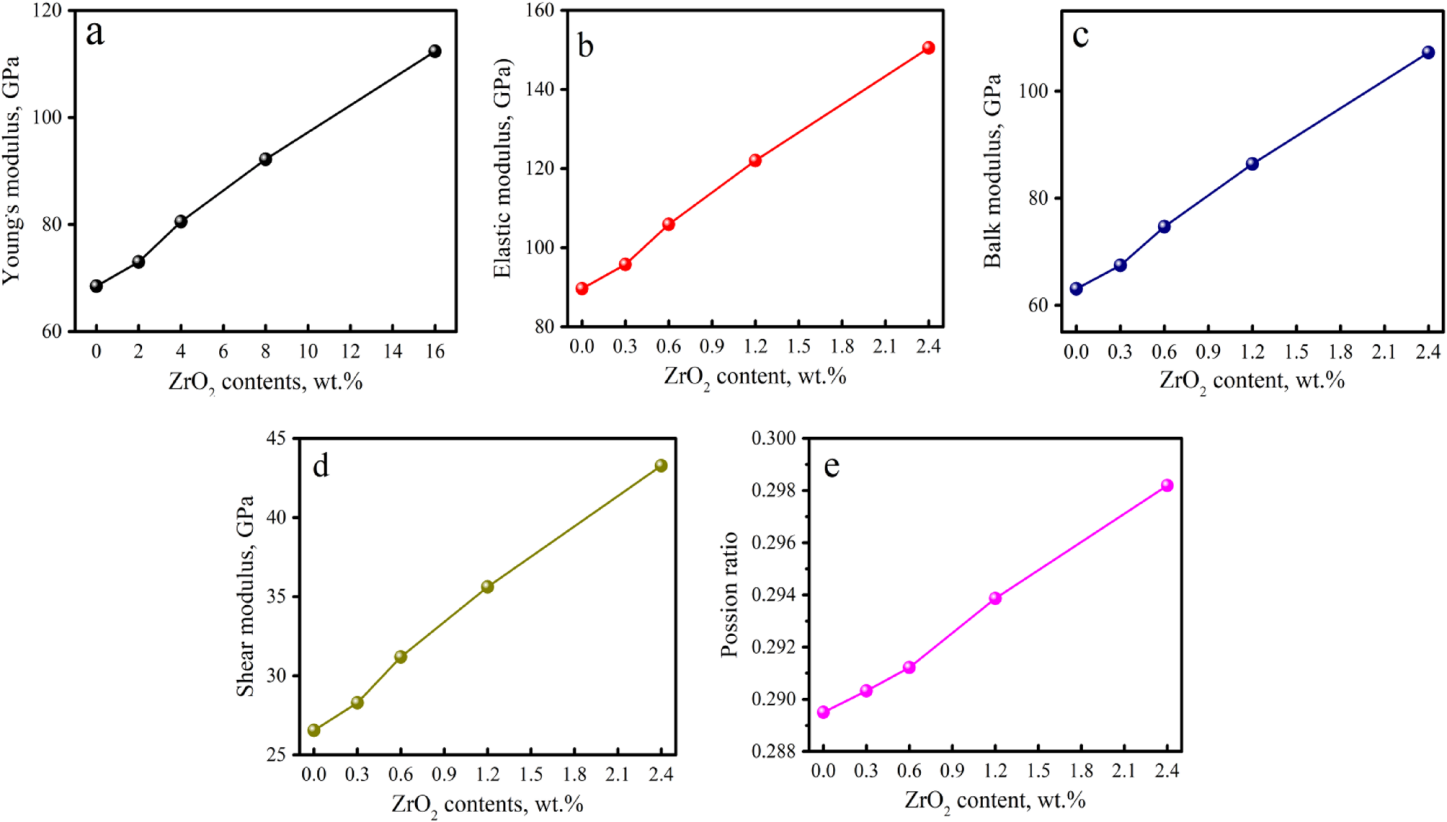
The group of elastic moduli of nanocomposites samples sintered at 570 °C in relation to varying ZrO_2_ weight percent.

As seen in Fig. 11, samples sintered at 570 °C were subjected to a non-destructive test (NDT) ultrasonic technique to evaluate the longitudinal (V_L_) and shear ultrasonic velocities (V_S_). It’s interesting to note that rising ZrO_2_ levels cause ultrasonic velocities to rise. The findings show that the samples’ V_L_ and V_S_ values range from 5886.4 to 7510.2 and 3205.6 4027.2 ms^–1^, respectively, with an increase in ZrO_2_ concentrations from 0 to 16 wt%. Figure 12 displays the elastic moduli of the studied nanocomposites. Moreover, the standard deviation of the measured ultrasonic velocities, and elastic modili are listed in Table 3. The figure makes it evident that the elastic moduli family has the same pattern for ultrasonic velocities. For instance, the elastic modulus and Poisson’s ratio in the AZ0 sample (i.e. the free content of ZrO_2_ particles) are 0.2895 and 89.6 GPa, respectively. Remarkably, they rise to 150.5 GPa and 0.2982, respectively, after 16 weight percent ZrO_2_ refinement (AZ16). Due to the addition of extremely hard nano-ZrO_2_ particles as reinforcement, ultrasonic velocities and elastic moduli significantly improved, entirely agreeing with the exact results of microhardness and compressive strength.

**Table 3:** The standard deviation of all mechanical properties for all samples tested.

Samples	Standard deviation						
	Longitudinal velocity	Shear velocity	Young’s modulus	Longitudinal modulus	Bulk modulus	Shear modulus	Poisson’s ratio
AZ0	10.8	15.4	0.55	0.34	0.33	0.26	0.002
AZ2	11.9	11.4	0.42	0.40	0.46	0.21	0.002
AZ4	14.8	16.8	0.71	0.55	0.57	0.34	0.003
AZ8	12.4	9.7	0.49	0.51	0.43	0.22	0.001
AZ16	17.1	10.1	0.53	0.72	0.64	0.23	0.001

Samples	Standard deviation						
	Microhardness	Ultimate strength	Yield strength	Elongation	Work hardening capacity		
AZ0	4.4	5.3	1.5	0.31	0.083		
AZ2	5.3	6.7	1.5	0.29	0.067		
AZ4	5.6	3.6	1.0	0.35	0.053		
AZ8	8.2	4.2	1.7	0.20	0.046		
AZ16	6.3	3.0	1.4	0.18	0.021		

**Figure 13:**
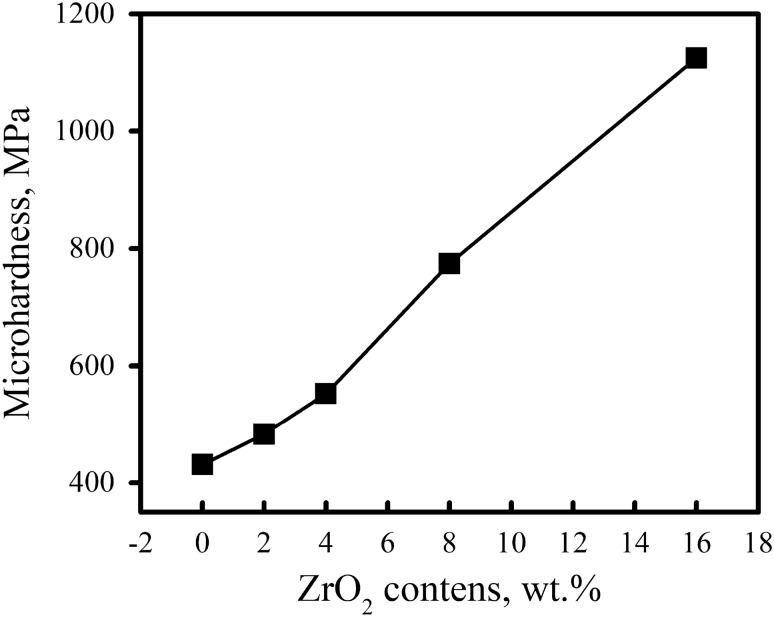
Microhardness of nanocomposites samples sintered at 570 °C.

Figure 13 displays the average microhardness values, or HV, of the Al alloy and Al alloy-ZrO_2_ nanocomposite samples that were sintered at 570 °C and the standard deviation listed in Table 3. The results show that there are noticeable increases in microhardness values when the amount of various ZrO_2_ particles in the Al alloy matrix rises. The addition of sixteen weight percent ZrO_2_ causes the microhardness of the Al alloy matrix to increase from 431.12 to 1124.5 MPa. The microhardness of nanocomposites samples can be explained by a number of factors, such as the presence of hard ceramic particles, the homogeneous distribution of reinforcement within the matrix, and the reduction of Al alloy matrix grain sizes with increasing ZrO_2_ content^39^.

**Figure 14:**
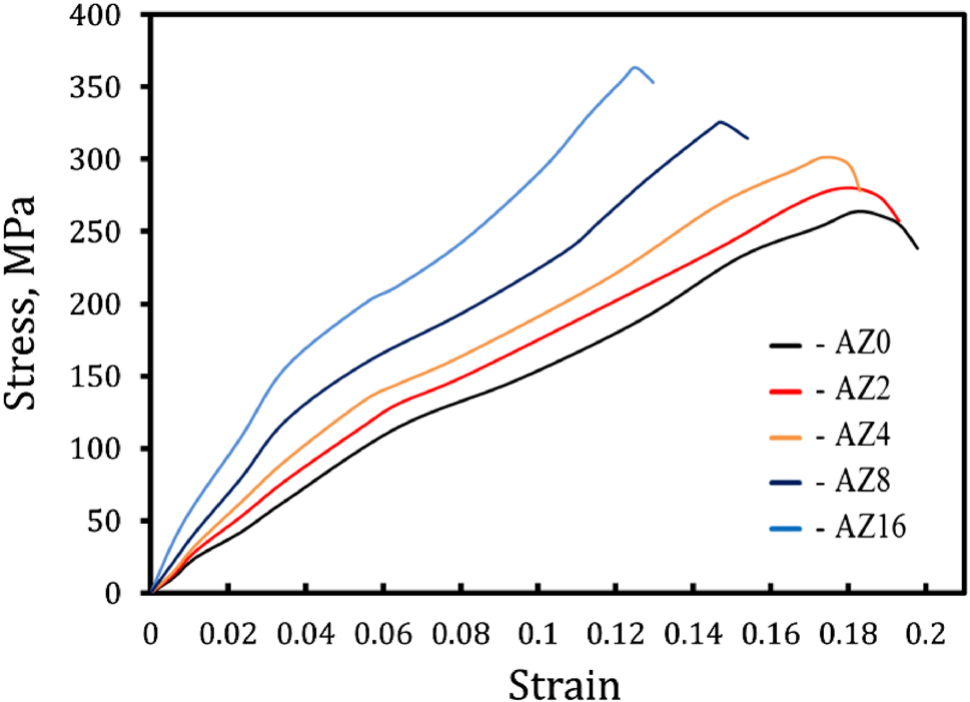
Compressive stress versus strain curve of Al alloy-ZrO_2_ samples sintered at 570 °C.

The samples’ compressive stress–strain curves are displayed in Fig. 14. It can be shown that Al alloy (AZ0) has a higher elongation and a lower yield end compressive strength than the other samples (AZ2, AZ4, AZ8, and AZ16) at the same sintering temperature. The values of ultimate compression strength (σ_ucs_), yield strength (σ_y_), and elongation (ε) were derived from the graphs produced from the compression tests of the AZ0, AZ2, AZ4, AZ8, and AZ16 nanocomposites samples. Moreover, the standard deviation of the measured σ_ucs_, σ_y_, and ε are listed in Table 3. These values are listed in Table 4. The findings indicate that when ZrO_2_ concentrations grow, elongation decreases, and both σ_ucs_ and σ_y_ of all the nanocomposites samples gradually increase. These findings are consistent with the trend in their microhardness results, as depicted in Fig. 13. For AZ0, the values of σ_ucs_, σ_y_, and ε are 263.82 MPa, 37.57 MPa, and 19.8%, respectively, whereas for AZ16, they were 383.46 MPa, 92.25 MPa, and 12.9%, respectively.

**Table 4:** The σ_ucs_, σ_y_, ε, and H_c_ of Al alloy-ZrO_2_ samples sintered at 570 °C.

Sample	σ_ucs_ (MPa)	σ_y_ (MPa)	ε (%)	H_c_
AZ0	263.82	37.57	19.8	6.02
AZ2	279.92	40.91	19.3	5.84
AZ4	301.26	47.81	18.3	5.30
AZ8	325.35	67.84	15.4	3.80
AZ16	363.46	92.25	12.9	2.94

Increases in ultimate and yield strength are often caused by a variety of causes, whereas the following parameters have an impact on the elongation of the Al alloy matrix reinforced with varying ZrO_2_ contents:

- (i) Thermal-mismatch strengthening

The significant discrepancy in the CTE of the ZrO_2_ particles and the Al alloy matrix is linked to thermal mismatch strengthening, which in turn leads to the creation of thermally induced residual stresses^38,39^. The thermal stresses produced in the Al alloy matrix, even at low temperatures, greatly increase the dislocation density close to the interface, strengthening the nanocomposite in the process.

- (ii) Orowan strengthening

The Orowan strengthening effect, which is caused by the uniform dispersion of the hard ZrO_2_ phase into the Al alloy matrix and serves as a barrier to dislocation movement, is essential in improving the mechanical properties of Al matrix nanocomposites. As a result, ZrO_2_ particles develop dislocation loops around them, increasing the tension needed to undergo further deformation.

- (iii) Load transfer from the Al alloy to the ZrO_2_ nanoparticles

In compressive testing, the load transfer, or σ_load_, between the hard ZrO_2_ particles and the Al alloy is determined by Eq. (3)^40^, particularly if there is a sufficient link between the reinforcement of ZrO_2_ particles and the Al alloy matrix.

3 $${\upsigma }_{{\text{load}}}=0.5{{\text{V}}}_{\mathrm{f }}{\upsigma }_{{\text{Ym}}},$$

where σ_Ym_ is the yield strength of the matrix.

It can be inferred that adding different ZrO_2_ amounts lowers the samples' work hardening capacity (H_c_). Equation (4) can be utilized to determine the H_c_ of nanocomposites samples by utilizing the values of σ_ucs_ and σ_y_.

4 $${\text{Hc}}= \frac{{\upsigma }_{{\text{ucs}}}-{\upsigma }_{{\text{y}}}}{{\upsigma }_{{\text{y}}}}.$$

The value of H_c_ for nanocomposites is represented in Table 4 and standard deviation is listed in Table 3 It is interesting to observe that the H_c_ of pressed nanocomposites decreases with an increase in the ZrO_2_ nanoparticle content. The nanocomposites' H_c_ depends on their yield strength, which is further correlated to grain sizes based on the Hall–Petch Eqn. If the grain sizes decrease, the difference in the flow resistance between the grain boundaries is also reduced, leading to an increase in the yield strength leading to decreased work hardening^41^.

#### Wear analysis

**Figure 15:**
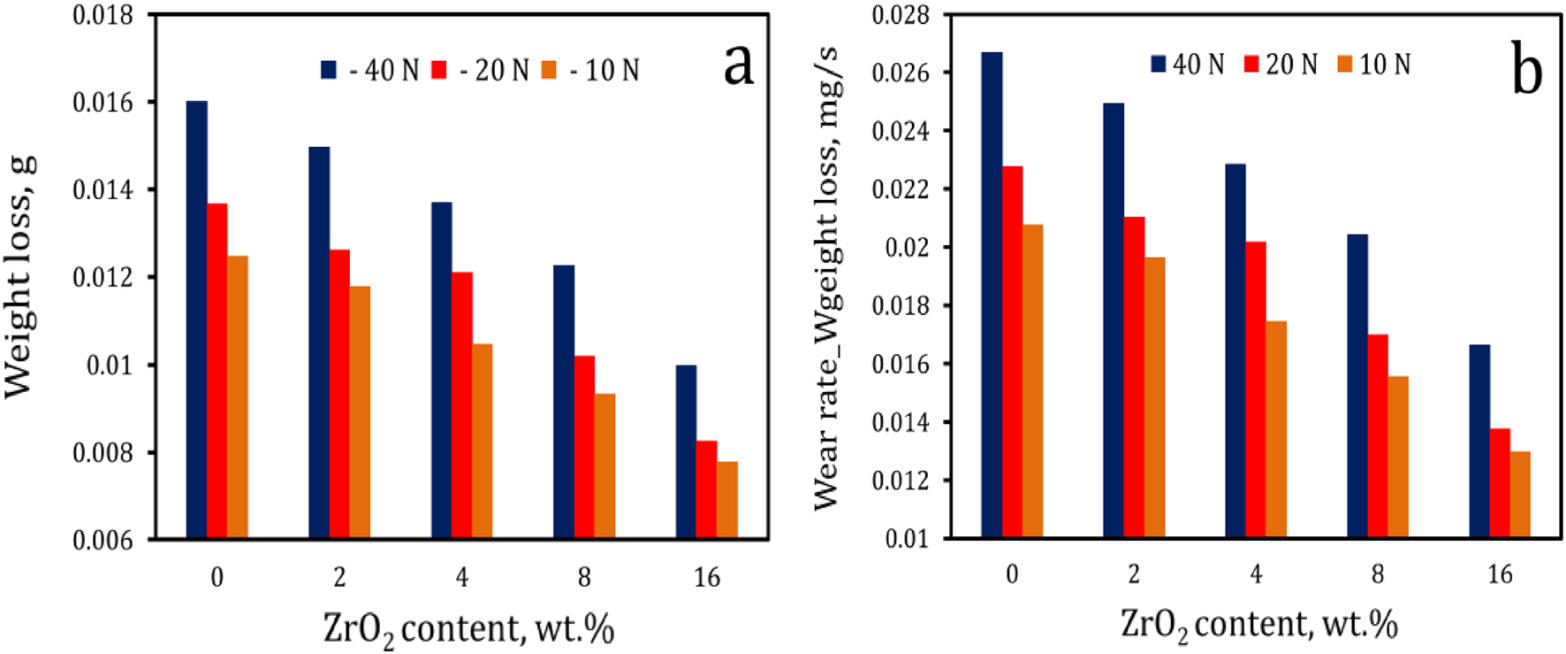
(**a**) Weight loss and (**b**) wear rate of sintered samples for different applied loads.

Figure 15 shows the variations in weight loss and wear rate of AZ0, AZ2, AZ4, AZ8, and AZ16 samples with different applied loads. Moreover, the standard deviation of the measured wear rate are listed in Table 5. The findings show that the ,wear resistance of nanocomposite samples tends to rise with increasing ZrO_2_ concentrations, while it decreases with increasing load. When applied stresses of 10, 20, and 40 N are used on an unreinforced sample (AZ0), the weight loss is 0.016, 0.0137, and 0.0125 g, respectively. The weight loss is 0.01 g, 0.0083 g, and 0.0078 g for the sample with 16 weight percent ZrO_2_ (AZ16) at the same applied loads. In addition, the wear rates of the nanocomposites samples AZ0, AZ2, AZ4, AZ8, and AZ16 are, respectively, 0.027, 0.025, 0.023, 0.02, and 0.017 mg/s when the applied stress is equivalent to 40 N. Wear resistance is shown to be significantly increased in the produced composites, which is unquestionably a benefit of the incorporation of ceramic particles (ZrO_2_)^42^. It is crucial to emphasize that adding ZrO_2_ particles to the Al alloy increases the nanocomposites' microhardness and strength, as was previously discussed. As a result, the wear rate decreases following Archad Eq. (5)^9^, which helps to explain the enhancement of wear resistance in nanocomposites.

**Table 5:** The standard deviation of wear rate for all samples tested.

Samples	Standard deviation		
	10 N	20 N	40 N
AZ0	0.00070	0.00039	0.00053
AZ2	0.00053	0.00067	0.00055
AZ4	0.00075	0.00059	0.00043
AZ8	0.00086	0.00029	0.00031
AZ16	0.00048	0.00067	0.00037

5 $${\text{W}}=\frac{\mathrm{k P}}{\mathrm{ H}}.$$

W is wear rate, K is a wear coefficient (constant value), P is the load, and H is the specimen’s Vickers hardness.

Furthermore, the increase in microhardness is consistent with a decrease in the real area of contact. It is well accepted that the real area of contact can be expressed in terms of the ratio of the normal load to the hardness of the pin material, and accordingly, the decreased real area of contact leads to considerable decreases in wear rate^43^. On the other hand, increases in weight loss and wear rate with increases in the applied load and the surface temperature encourage surface softening, causing more surface and subsurface damage and resulting in decreased wear resistance^44^.

### Corrosion analysis

**Figure 16:**
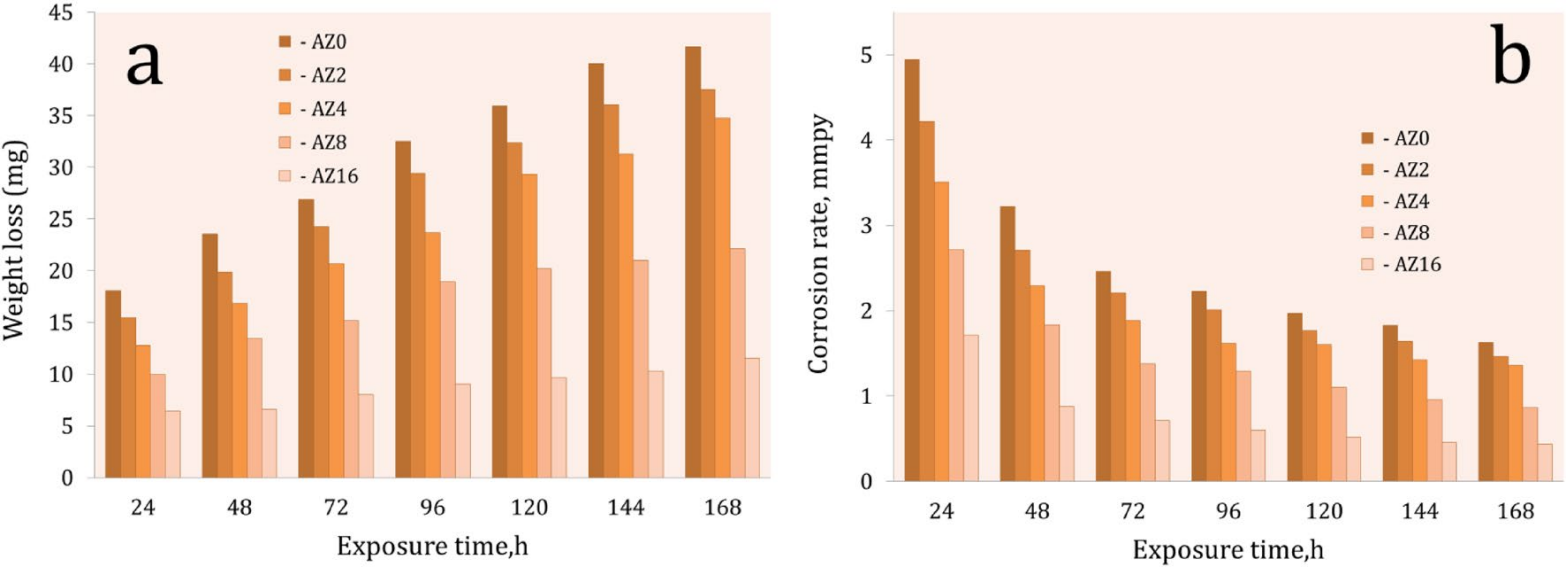
(**a**) Weight loss and (**b**) corrosion rate of sintered samples for different applied loads.

The corrosion behavior of Al or nanocomposites specimens in an acidic medium was assessed using the weight-loss method. The corrosion behavior of the nanocomposites under study is often influenced by a wide range of parameters, including weight percentages of ZrO_2_ reinforcement, compaction, density, and sintering. This means that after being submerged in 0.1N HCl at room temperature (30 °C), the weight loss and corrosion rate of the AZ0, AZ2, AZ4, AZ8, and AZ16 sintered samples were measured and shown in Fig. 16. Furthermore, the standard deviation of the measured corrosion rate are listed in Table 6. It is noteworthy to note that as exposure time increases, Al alloy matrix nanocomposites lose weight more quickly, which lowers the rate of corrosion. The sample loses weight as exposure time increases because the rate of corrosion reduces with longer contact with an acidic liquid^44^. When the AZ0 sample is immersed for 24, 96, and 168 h, its weight loss is 18.04, 32.52, and 41.65 mg, respectively. When the AZ16 sample, which contains 16 percent ZrO_2_, is immersed for the same amount of time, its weight loss is 6.43, 9.06, and 11.52 mg, respectively. It was also discovered that as ZrO_2_ levels increase, weight loss and corrosion rates decrease. Since the ceramic reinforcement particles often don’t exhibit any discernible corrosive activity, the presence of ZrO_2_-particles on the surface of nanocomposites samples will keep the surface layer safe in acidic environments^45–47^. The corrosion rate of the AZ0 sample is higher than that of the AZ16 nanocomposite samples. This result can be attributed to the high corrosion resistance of ZrO_2_, a ceramic material that resists corrosion well enough to be inert and unaffected by the acidic medium during corrosion tests. After being submerged for 168 h, the corrosion rates of the AZ0, AZ2, AZ4, AZ8, and AZ16 samples are 1.63, 1.46, 1.36, 0.86, and 0.44 mmpy, in that order.

**Table 6:** The standard deviation of corrosion rate for all samples tested.

Samples	Standard deviation						
	24 h	48 h	72 h	96 h	120 h	144 h	168 h
AZ0	0.054	0.032	0.014	0.013	0.01	0.009	0.01
AZ2	0.058	0.026	0.011	0.012	0.008	0.01	0.007
AZ4	0.033	0.011	0.012	0.009	0.009	0.007	0.08
AZ8	0.028	0.009	0.008	0.01	0.011	0.004	0.004
AZ16	0.009	0.007	0.004	0.005	0.006	0.005	0.003

## Conclusions

In the current study, Al-Cu-Mg alloy-ZrO_2_ nanocomposites were prepared using powder metallurgy; the following conclusions were drawn:

- Observations were made that the nanocomposites prepared by the powder metallurgy process had a good distribution of nano-ZrO_2_ particles in a matrix with noticeable agglomerations.
- The particle size reduced with increasing the ZrO_2_ particles until they reached 45.8 nm for the sample containing 16 wt.% ZrO_2_.
- The relative density of the samples decreased with increasing ZrO_2_ weight percent, while the apparent porosity increased.
- The measurement of thermal expansion reflected that the CTE of the Al alloy was decreased by about 28.2% with the addition of 16 wt.% ZrO_2_ particles, indicating the high dimensional stability of nanocomposite samples.
- Elastic moduli increased when ZrO_2_ quantities increased because the sintered samples' ultrasonic velocities increased as well. Adding 16 weight percent of ZrO_2_ improved the bulk and elastic moduli to 70 and 68 percent, respectively.
- As the weight percentages of ZrO_2_ were increased, the microhardness, ultimate, and yield strength were improved, but the elongation and work hardening were decreased. The AZ16 sample exhibited maximum values for microhardness and ultimate strength, which were found to be approximately 1.8 and 2.1 times greater, respectively, compared to the AZ0 sample.
- Specimen wear rates rose with increased load but decreased with increasing ZrO_2_. By adding 12 weight percent ZrO_2_ particles, the wear rate of the Al alloy was reduced by about 25% compared to the Al alloy matrix.
- It has been discovered that raising the weight percentages and exposure times of ZrO_2_ particles helps to decrease the rate of corrosion of nanocomposites.

## Data Availability

The datasets generated and/or analyzed during the current study are not publicly available because all data are presented in the article and therefore, there is no need to include raw data but they are available from the corresponding author upon reasonable request.
